# Toward Accurate Two-Photon Absorption Spectrum Simulations: Exploring the Landscape beyond the Generalized Gradient Approximation

**DOI:** 10.1021/acs.jpclett.3c03513

**Published:** 2024-01-22

**Authors:** Karan Ahmadzadeh, Xin Li, Zilvinas Rinkevicius, Patrick Norman, Robert Zaleśny

**Affiliations:** † Division of Theoretical Chemistry and Biology, School of Engineering Sciences in Chemistry, Biotechnology and Health, KTH Royal Institute of Technology, SE-100 44 Stockholm, Sweden; ‡ PDC Center for High Performance Computing, KTH Royal Institute of Technology, SE-100 44 Stockholm, Sweden; ¶ Department of Physics, Faculty of Mathematics and Natural Sciences, Kaunas University of Technology, Kaunas LT-51368, Lithuania; § Faculty of Chemistry, Wrocław University of Science and Technology, Wyb. Wyspiańskiego 27, PL-50370 Wrocław, Poland

## Abstract

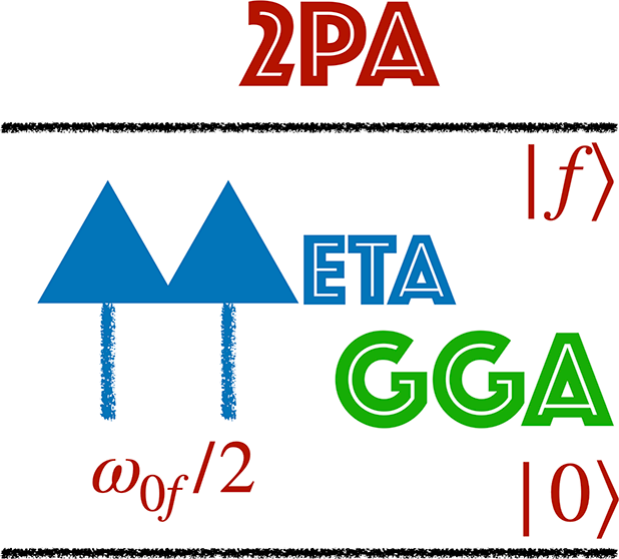

In this Letter, we present a pioneering analysis of the density functional approximations (DFAs) beyond the generalized gradient approximation (GGA) for predicting two-photon absorption (2PA) strengths of a set of push–pull π-conjugated molecules. In more detail, we have employed a variety of meta-generalized gradient approximation (meta-GGA) functionals, including SCAN, MN15, and M06-2X, to assess their accuracy in describing the 2PA properties of a chosen set of 48 organic molecules. Analytic quadratic response theory is employed for these functionals, and their performance is compared against the previously studied DFAs and reference data obtained at the coupled-cluster CC2 level combined with the resolution-of-identity approximation (RI-CC2). A detailed analysis of the meta-GGA functional performance is provided, demonstrating that they improve upon their predecessors in capturing the key electronic features of the π-conjugated two-photon absorbers. In particular, the Minnesota functional MN15 shows very promising results as it delivers pleasingly accurate chemical rankings for two-photon transition strengths and excited-state dipole moments.

Two-photon absorption (2PA) is a phenomenon employed in an array of technological applications, such as 3D microfabrication^1^ and multiphoton imaging.^2^ The prediction of the 2PA strengths is essential for the development and optimization of novel functional materials.^3,4^ Algorithms for theoretical calculations of 2PA strengths have been implemented at various levels of theory,^5,6^ including time-dependent density functional theory (TD-DFT).^7−9^ The accuracy of the predicted two-photon absorption strengths from TD-DFT will depend on the exchange–correlation functional and its higher-order derivatives. The excitation energies are obtained from the linear response and depend on the electronic Hessian and therefore second-order derivatives of the exchange–correlation functional with respect to variations of the density. The two-photon absorption tensors require evaluation of the corresponding third-order derivatives. For linearly polarized light, the rotationally averaged two-photon absorption strength is given in terms of the two-photon transition moments:^10^
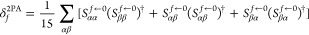
1where
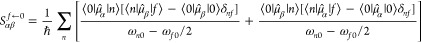
2The two-photon transition moments are in turn calculated from the residue of the quadratic response function:
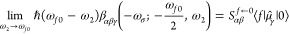
3

In TD-DFT, the evaluation of the residue of the quadratic response function requires the third-order derivative of the exchange–correlation functional or the second-order nonlinear exchange–correlation kernel. During the last two decades, a number of groups have attempted to assess the performance of DFAs in simulations of 2PA spectra and various coupled-cluster models were used as reference.^11−14^ It should be noted that 2PA strengths calculated with the CC2 method have been benchmarked against higher-order coupled-cluster methods for very small organic molecules, and it was demonstrated that CC2 overestimated some 2PA strengths by up to factor of 2 compared with CC3.^15^ However, some other works performed for medium-sized organic molecules demonstrated that the discrepancy between CC2 and higher-order CC models might be much smaller (up to factor 1.4 depending on the system).^13^ A recent study by Andruniów’s group demonstrated that the differences between CC2 and CCSD/CC3 can be even smaller for medium-sized organic chromophores, and we find this result as very relevant for the present Letter, given the fact that we also study π-conjugated molecules.^16^ Another study demonstrated that 2PA strengths for charge-transfer systems showed good qualitative agreement between CC2 and CAM-B3LYP for a weakly bound tweezer complex.^17^ A larger benchmark study has also been performed on a set of 48 charge-transfer systems, predominantly focused on pure, hybird, and range-separated functionals belonging to the GGA class of functionals.^18^ This study revealed that range-separated GGAs, such as CAM-B3LYP and optimally tuned LC-BLYP, can provide strong linear correlations with CC2 2PA strengths in π-conjugated charge-transfer molecules containing electron-donating/accepting moieties, albeit severely underestimating 2PA strengths. In contrast, it was also shown that global hybrid GGAs were more successful in reproducing absolute 2PA strengths for many donor–acceptor molecules but exhibited worse Pearson coefficient values compared to range-separated functionals. Moreover, a generalized-few-state model analysis revealed that global hybrid GGAs tend to deliver good results for the wrong reasons.^18^ This benchmark study also demonstrated that none of the studied functionals provide accurate absolute 2PA strengths from the residue of the quadratic response function, and approximations to the 2PA strengths in the form of a few-state model can be helpful in pinpointing the origin of the observed discrepancies. In more detail, the summation in the expression for the two-photon transition moment tensor can be truncated to only involve two states yielding the approximate two-state model (2SM) to estimate of the two-photon absorption strength:^18^
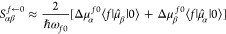
4and
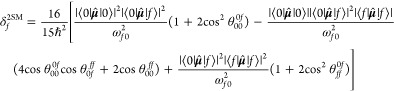
5where the introduced angles are those found in between the various dipole moment and transition dipole moment vectors. The excited state dipole moment appearing in the above equations are obtained from the double residue of the quadratic response function:
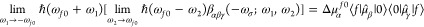
6where *Δμ*^*f*0^ denotes the excited to ground state dipole moment difference. Note that the evaluation of the double residue of the quadratic response function will also depend on the second-order nonlinear exchange–correlation kernel. Provided that differences between key excited- and ground-state dipole moments are underestimated, the corresponding 2PA strength evaluated based on the residue of the quadratic response function might also be affected in a similar way.^13^ The systematic underestimation of 2PA strengths observed from approximate DFT functionals has been understood as being due to the overestimation of the ground-state dipole moment and the underestimation of the excited-state dipole moment.^19^ In a comparative benchmark study of density functionals for the estimation of excited-state dipole moments on a set of photochromic molecules, it was shown that none of the employed exchange–correlation functionals in that study could capture the magnitude of the excited-state dipole moments correctly when compared to CC2.^20^ The poor estimation of the excited-state dipole moments and 2PA strengths in the above-mentioned studies indicates that the use of new classes of functionals is in demand to achieve accurate values of 2PA strengths and high correlation coefficients (chemical ranking).

The unsatisfactory performance of hybrid and range-separated hybrid functionals motivates the present study, which reports on the pioneering exploration of 2PA strengths obtained based on meta-GGA functionals. We find this study to be very timely following our recent implementation in the VeloxChem program^21^ of the exchange–correlation kernel required to perform quadratic response calculations with meta-GGA functionals. This development and the use of LibXC library^22^ enable the calculations of 2PA strengths with meta-GGA functionals for the fist time, and the aim of this Letter is to assess whether the meta-GGA functionals show any promise in improving predictions of 2PA strengths.

To that end, a set of 48 medium-sized donor−π–acceptor molecules are investigated using the single-reference coupled-cluster CC2 method (RI-CC2) and a selection of DFAs, including empirically parametrized, nonempirically parametrized, pure, and hybrid functionals. The very same set of molecules was studied recently by Chołuj et al.,^18^ and we refer readers to this work for chemical structures. In what follows, we will analyze the lowest ππ*-excited states of intramolecular charge-transfer character as being due to the electron-donating and electron-accepting moieties. These states are found to be either *S*_1_ or *S*_2_ in the calculations. In this Letter, we employ the newly implemented quadratic response at the meta-GGA level of theory (computational details are presented in the Supporting Information) and compare the performance of the meta-GGA DFAs with those of the previously studied GGA DFAs and the RI-CC2 model used as a reference. A detailed analysis of the meta-GGA functional performance is provided, focusing on their ability to capture the key electronic and structural features of the studied π-conjugated systems.

We will start with an analysis of 2PA strengths in terms of chemical ranking. The Pearson correlation with respect to RI-CC2 for the two-photon absorption strength calculated using the residue of the quadratic response function for the whole data set is presented in Figure 1.

**Figure 1:**
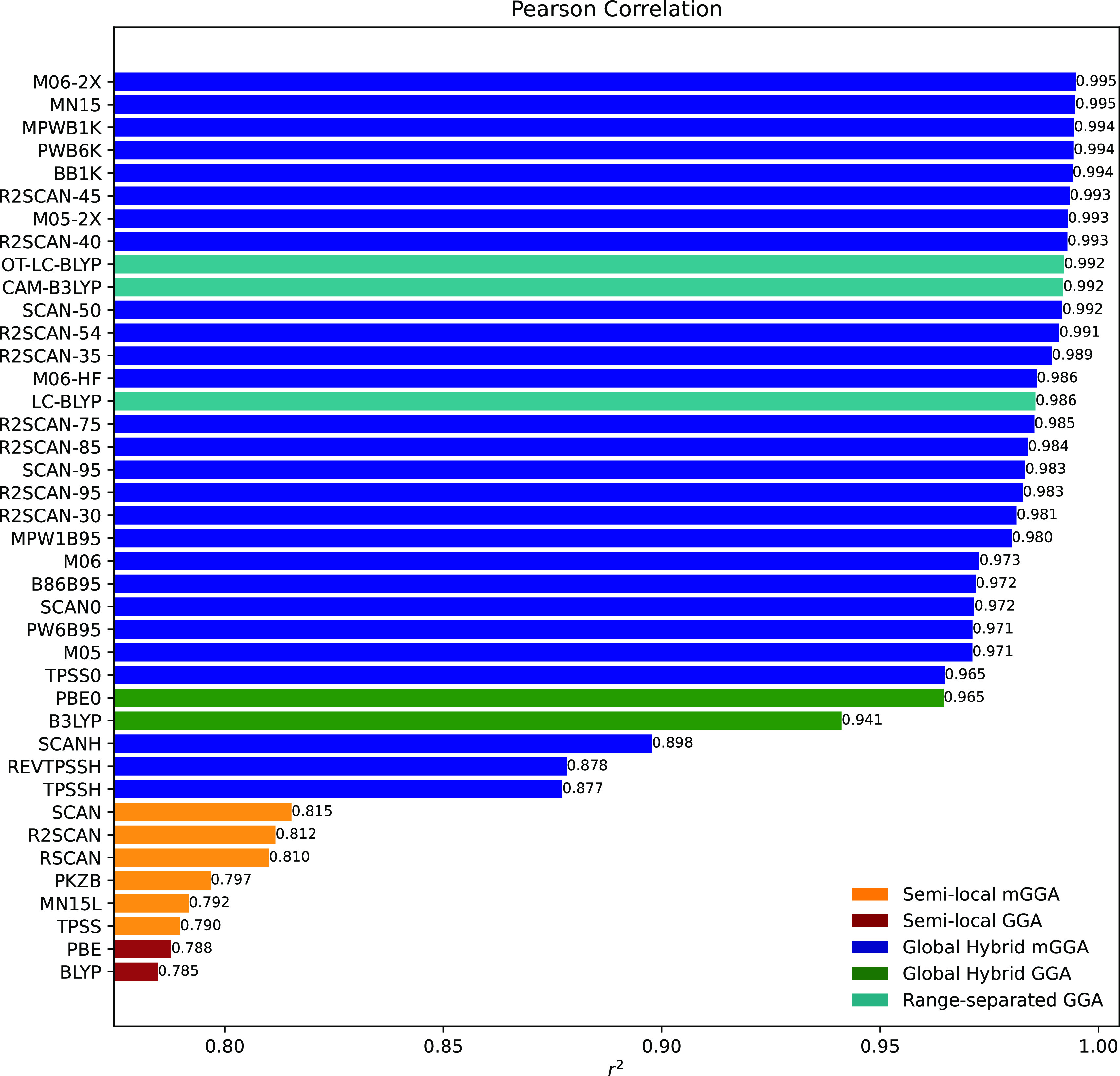
Pearson correlation coefficients (PCCs) for the 2PA strengths at the DFT level of theory for molecules 1–48 with RI-CC2 as reference.

In terms of Pearson (and Spearman, see the Supporting Information) correlation coefficients, all of the semilocal functionals deliver values below 0.85, which would be expected considering the charge-transfer character of the excitations. To alleviate the incorrect asymptotic behavior of the semilocal functionals, some fraction of exact exchange must be added. An improvement in the correlation coefficients can be observed for the global hybrids and range-separated functionals, which all yielded correlation coefficients above 0.85. A known tendency for this set of molecules from previous studies is that the long-range corrected functionals yielded the best linear correlation coefficients but, on the other hand, global hybrids were more successful in reproducing the absolute values of 2PA strengths.^18^ To get a more comprehensive perspective on this data one therefore also needs to consider the relative errors, which are shown in Figure S4 and Table S1. Taking both correlation coefficients and relative errors into consideration, we see that the best semilocal GGA was PBE with a relative error of 129% and Spearman and Pearson correlations of 0.77 and 0.79, respectively. The best global hybrid GGA was PBE0 with a mean relative error of around 39% and Spearman and Pearson correlation coefficients of 0.87 and 0.96. The global hybrid meta-GGAs MN15 and MPWB1K gave comparable Spearman and Pearson correlations to the best long-range corrected functional CAM-B3LYP; however, the former two functionals showed significantly lower mean relative errors than the latter one. Looking at the distribution of errors as can be seen in Figure S4, MN15 has roughly 79% of its relative errors below 50% as compared to CAM-B3LYP, which had comparable correlation coefficients but only 39% of its relative errors below 50%. Comparing MN15 and MPWB1K with PBE0 in Figure S4, we see that PBE0 has roughly double the standard deviation and more extremes in its relative error profile. These results indicate that the mentioned meta-GGA functional seems to combine the best of both worlds, having correlation coefficients similar to the best long-range corrected functional while still reproducing comparable or more consistent 2PA strengths than the global hybrid GGA functionals previously studied. Given the high Pearson correlation observed for MN15 and the popularity of the M06-2X functional in spectroscopic studies, we will include them in further in-depth analyses. Finally, it is fair to mention that many other meta-GGA functionals are as good as CAM-B3LYP for chemical rankings.

The performance of selected global hybrid meta-GGA and long-range corrected GGA functionals in predicting 2PA strengths for the whole set of 48 molecules is shown in Figure 2. One clear trend that is consistent with previous studies of this set of push–pull π-conjugated systems is that DFAs systematically underestimate the 2PA strengths (except MN15 in a handful of cases).

**Figure 2:**
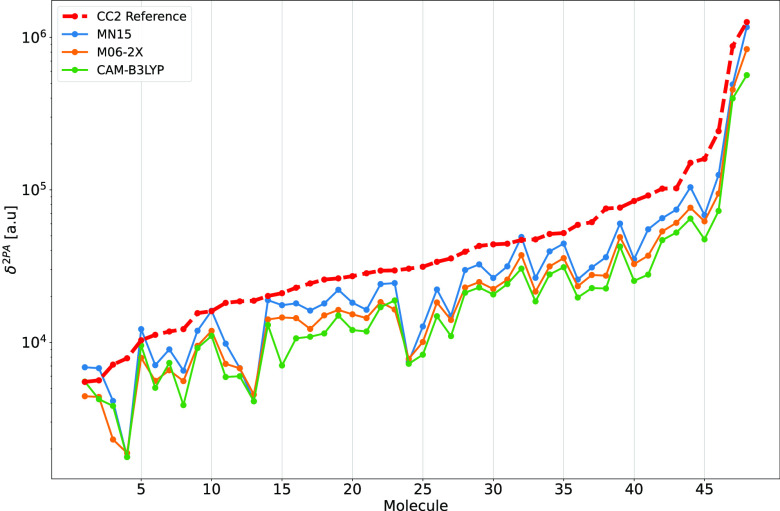
Two-photon absorption strengths computed using the RI-CC2 method and density functional approximations for the whole set of molecules.

As highlighted in preceding paragraphs, the main reason for the underestimation of the 2PA strengths by range-separated DFAs was attributed to the underestimation of the difference between the dipole moments of the ground and excited states.^18^ The results for the best global-hybrid meta-GGA DFAs also follow this trend, as can be seen in Figure 3. We note that MN15 and M06-2X, which are among the best DFAs in terms of correlation coefficients and mean relative errors, still underestimate the difference in dipole moment between ground and excited states, albeit to a lesser extent than the best long-range corrected DFA, CAM-B3LYP (Figure 3). Similar conclusions regarding M06-2X and MN15 performances in predictions of dipole moment differences were presented by Jacquemin^23^ and Grabarz et al.,^24^ respectively. The difference between the excited- and ground-state dipole moments shown in Figure 3 applies to the excitation path dominant in the 2PA process. The dominance of this transition pathway can be confirmed by comparing the 2PA strengths from response theory (RSP) and 2SM in Figure S2, where the RSP and 2SM trends are qualitatively very similar, indicating that the 2PA strength has a dominant excitation pathway. The underestimation of the 2SM 2PA strengths can be explained by looking at Figure 3 and Figure S3, i.e., it can be seen that CAM-B3LYP underestimates the dipole moment of the excited state and in most cases overestimates the excitation energies. Both errors lead to a prediction of the underestimated 2PA strengths. Interestingly, M06-2X delivers very similar results for the excitation energies as CAM-B3LYP for this set of systems presenting intramolecular charge transfer upon excitation to low-lying ππ*-states, while MN15 performs comparatively worse in this metric compared to its performance in the other cases (for more extensive data on predictions of excitation energies we refer to Figures S7 and S8 and Table S3). Thorough analyses of DFA performance in computing excitation energies can be found elsewhere.^25−30^

**Figure 3:**
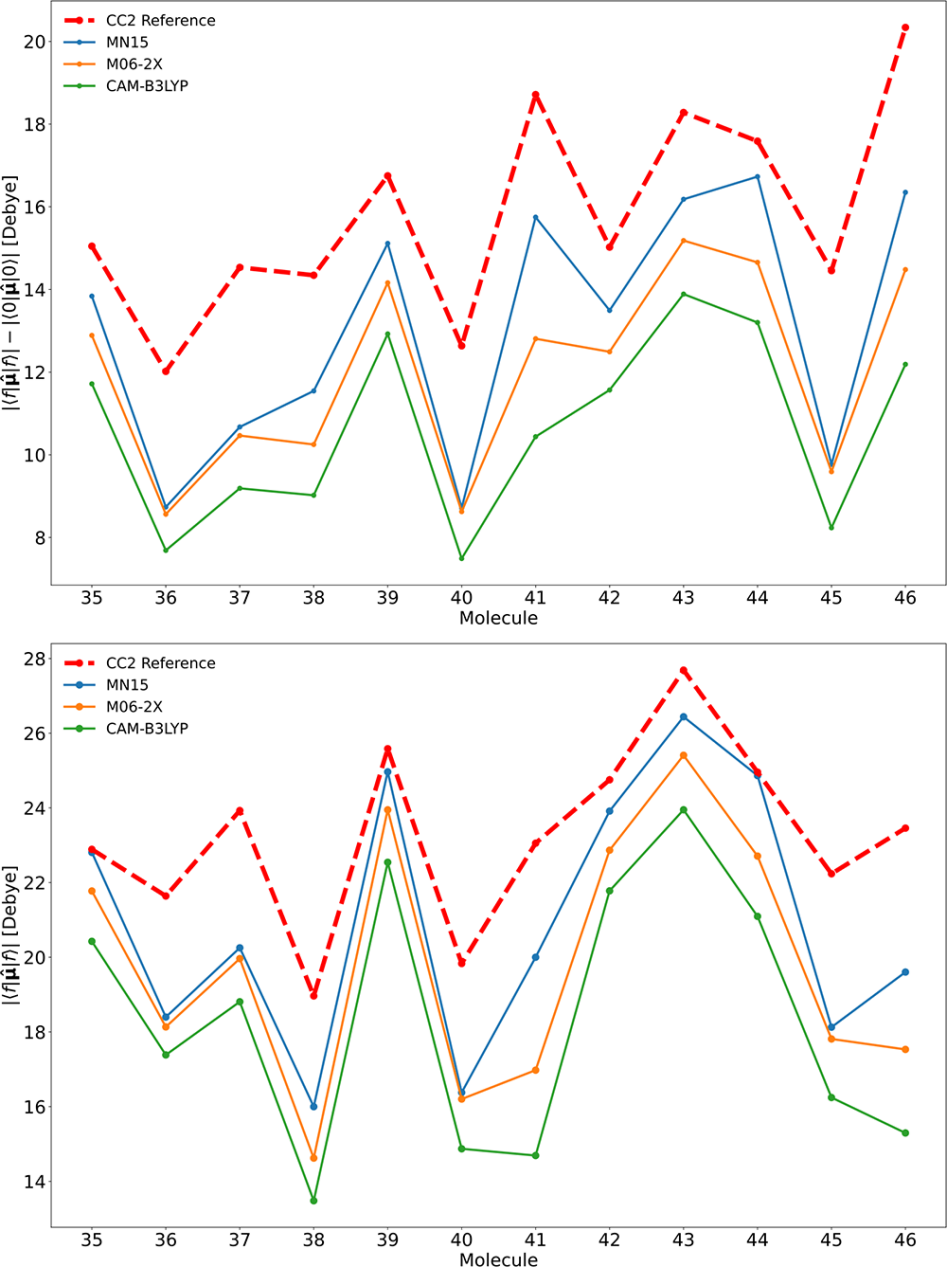
Difference between the excited-state and ground-state dipole moments (top) and excited-state dipole moments (bottom) for molecules 35–46 for a selection of global hybid meta-GGA functionals and a long-range corrected GGA functional with the RI-CC2 level as reference.

Figure S5 shows the Pearson correlation coefficients for the excited-state dipole moments as compared with RI-CC2. The following assessments are made: the best semilocal GGA is found to be PBE with a Spearman and Pearson correlation of 0.6 and 0.21 with a mean relative error of 13.1%; the best semilocal meta-GGA is SCAN with Spearman and Pearson correlations of 0.63 and 0.25 and a mean relative error of 15%; the best global-hybrid GGA is PBE0 with Spearman and Pearson correlations of 0.74 and 0.6 and a mean relative error of 10.8%; the best global-hybrid meta-GGA is BB1K with Spearman and Pearson correlations of 0.92 and 0.86 and a mean relative error of 8.9% followed by MPWB1K and MN15; and the best long-range corrected GGA is CAM-B3LYP with Spearman and Pearson correlations of 0.83 and 0.73 and a mean relative error of 21.3%. In view of Figure S6, it is seen that MN15 had a more favorable error distribution as compared to PBE0 and CAM-B3LYP. Most of the studied global-hybrid meta-GGA functionals with a fraction of exact exchange in the range between 0%–54% did well in terms of Pearson and Spearman correlations. Overall, it follows from our analysis that the strong point of meta-GGA functionals is that they improve predictions of excited-state dipole moments upon range-separated functionals. In this context, let us mention recent work of Knysh et al., who studied excited-state dipole moments of push–pull oligomers of different sizes.^31^ These authors demonstrated good agreement between EOM-CCSD and CC2 up to seven vinylene units. In the case of longer chains, the CC2 tends to overestimate the excited-state dipole moments.^31^ Taken together, the results by Knysh et al.^31^ support the choice of CC2 method as reference for the present analysis.

Finally, in Figure S9, we show the effect of the amount of exact exchange on the two-photon absorption strength and the various associated electronic-structure parameters. As the amount of exact exchange increases, the predicted 2PA strength tends to decrease. This trend can be anticipated based on the excitation energies and the difference in dipole moments of the excited and ground states. An increase in the exact exchange leads to an underestimation of the dipole moment difference and an overestimation of the excitation energies. Both errors lead to an underestimation of the 2PA strength, which is also reported in the present Letter. However, the transition dipole moments tend to increase with the fraction of the exact exchange.

In summary, the performance of a large set of DFAs, including for the first time the meta-GGA class of functionals, for the calculation of two-photon absorption strengths was investigated for a set of 48 push–pull π-conjugated molecules. The focus was put on electronic transitions of intramolecular charge-transfer character. Previous studies on the performance of GGA functionals in the calculation of 2PA strengths have shown a systematic underestimation of this property, which has been attributed to an underestimation of the difference between the excited and ground-state dipole moments. In this study, we have compared a large set of global hybrid meta-GGA functionals with previously studied global and range-separated GGA functionals, and we have shown that the global hybrid meta-GGA functionals, e.g., MN15, MPWB1K, and BB1K, have similar Pearson and Spearmann correlations with respect to RI-CC2 as range-separated GGAs such as CAM-B3LYP and OT-LC-BLYP, while having much lower mean relative errors. An analysis of the excited-to-ground state dipole moment difference for a subset of the molecules showed that the mentioned meta-GGA functionals still underestimate the magnitude of the dipole moment difference, albeit to a lesser extent than the range-separated GGA functionals. Furthermore, we have investigated the role of the fraction of exact exchange in global hybrid functionals on the predicted 2PA strengths. We show that higher fractions of exact exchange lead to systematically lower predictions of the magnitude of the difference between the excited- and ground-state dipole moments and overestimations of excitation energies resulting in underestimations of 2PA strengths. With molecular design of two-photon absorbing materials in mind, we recommend the usage of the Minnesota functional MN15 as it delivers pleasingly accurate chemical rankings for two-photon transition strengths and excited-state dipole moments.

## Supporting Information Available

Additional results of electronic-structure calculations for molecules 1–48 (PDF)
