# Supplementary material for: Toward Accurate Two-Photon Absorption Spectrum Simulations: Exploring the Landscape beyond the Generalized Gradient Approximation
Source: J Phys Chem Lett. 2024 Jan 22;15(4):969–74. doi: 10.1021/acs.jpclett.3c03513 (PMC10839899; doi:10.1021/acs.jpclett.3c03513)
Supplement: Supplementary file 1 — jz3c03513_si_001.pdf [file jz3c03513_si_001.pdf]

Supporting Information: Toward Accurate Two-Photon Absorption Spectrum Simulations: Exploring the Landscape Beyond the Generalized Gradient Approximation

Karan Ahmadzadeh,[*,†] Xin Li,[‡] Zilvinas Rinkevicius,[†,¶] Patrick Norman,[*,†] and Robert Zaleśny[*,§]

† *Division of Theoretical Chemistry and Biology, School of Engineering Sciences in Chemistry, Biotechnology and Health, KTH Royal Institute of Technology, SE-10044 Stockholm, Sweden*

‡ *Center for High Performance Computing, KTH Royal Institute of Technology, SE-100 44 Stockholm, Sweden*

¶ *Department of Physics, Faculty of Mathematics and Natural Sciences, Kaunas University of Technology, Kaunas LT-51368, Lithuania.*

§*Faculty of Chemistry, Wrocław University of Science and Technology, Wyb. Wyspiańskiego 27, PL-50370 Wrocław, Poland*

E-mail: karana@kth.se; panor@kth.se; robert.zalesny@pwr.edu.pl

Computational details

All response-theory calculations were performed using VeloxChem[S1] and the def2-SVPD basis set with a grid level of 6 (as defined in VeloxChem program). The convergance threshold for the response vectors was set to 1.0×10^{-4}.

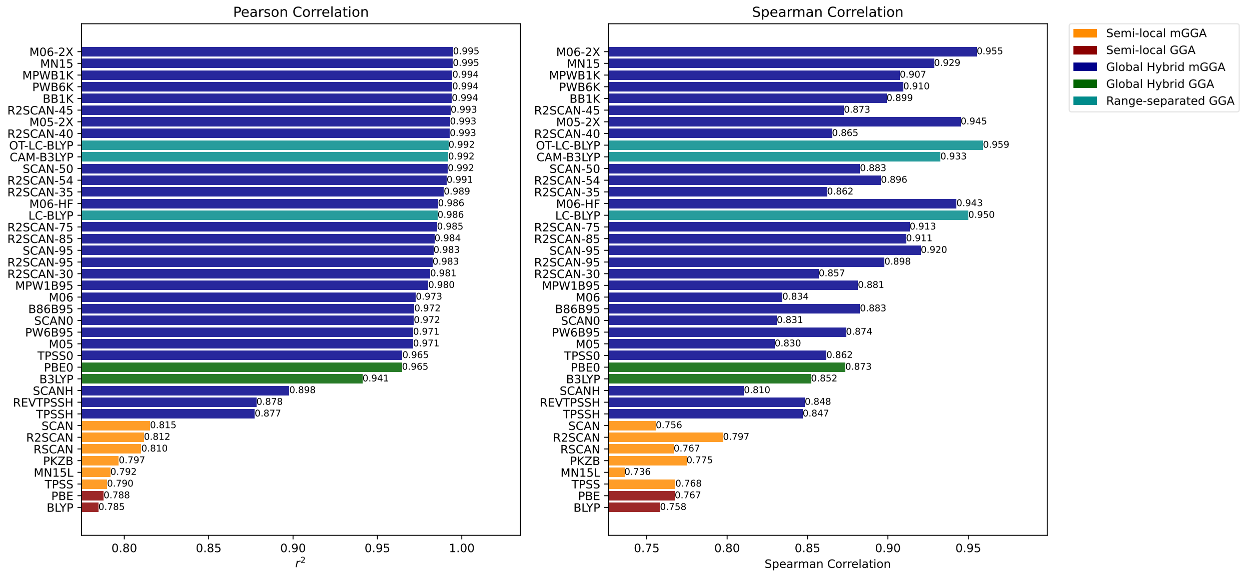

Figure S1: Pearson-Correlation (r^2) and Spearman-Correlation for the calculation of TPA strengths from the residue of the quadratic response function at the DFT level of theory for molecules 1-48 with RI-CC2 as reference.

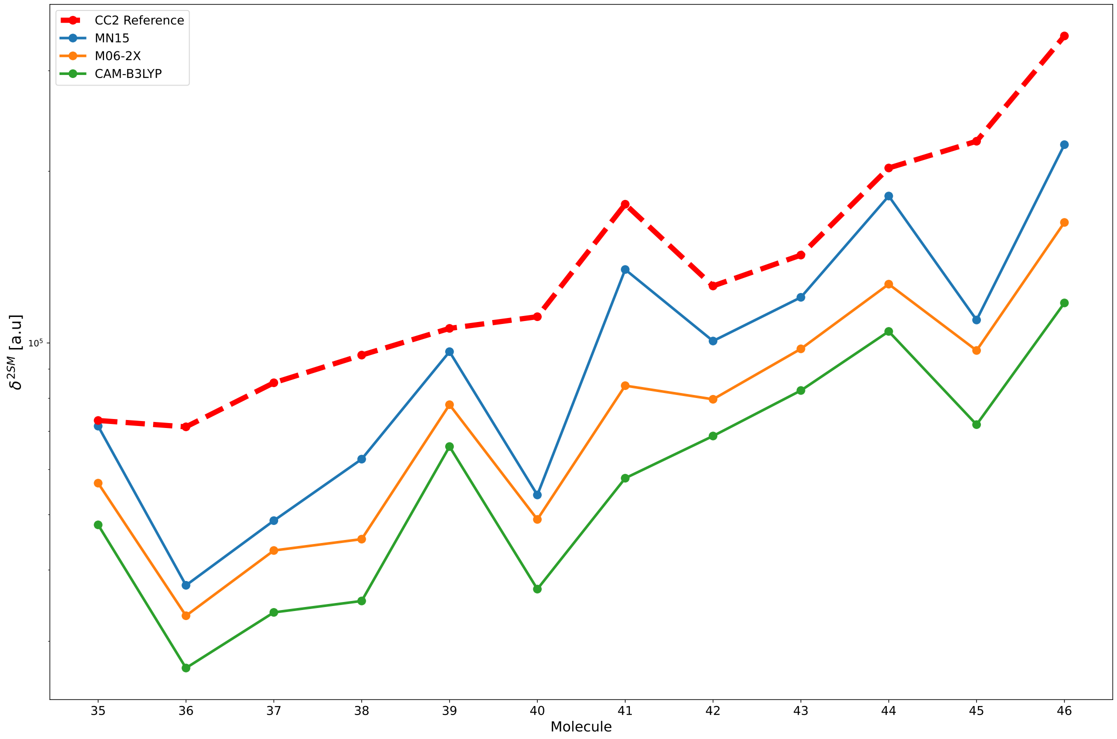

(a)

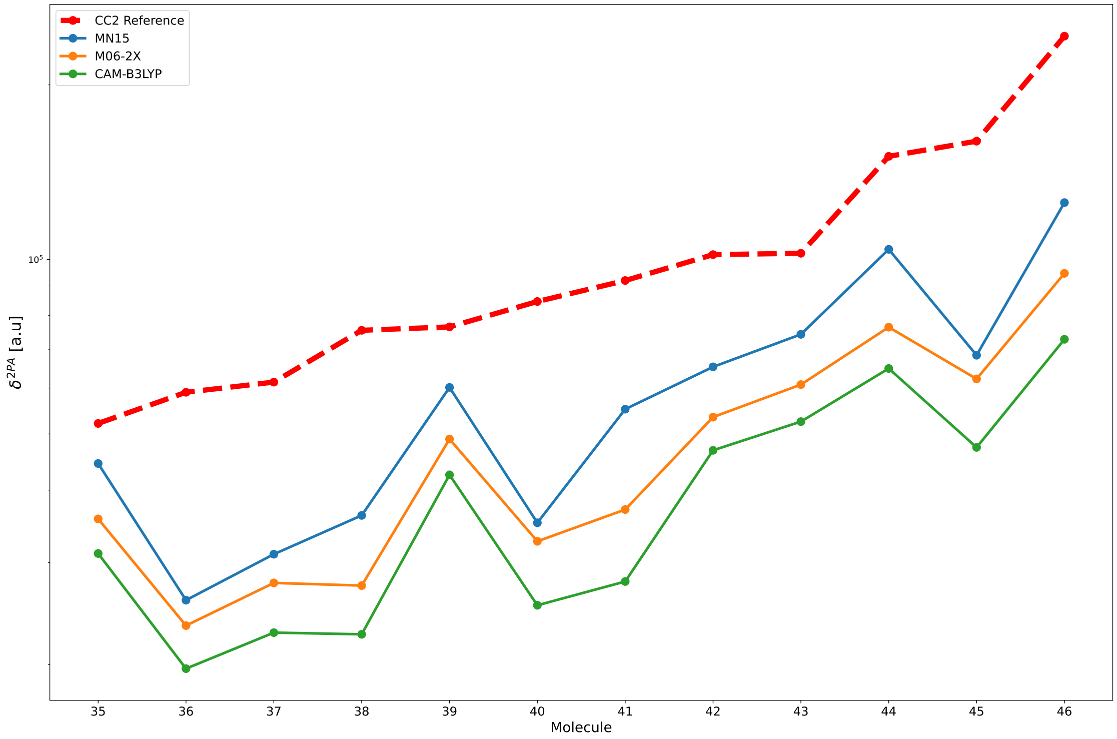

(b)

Figure S2: Comparison of the two-state model a) and RSP b) 2PA strengths for selected global meta-GGA functionals and a long-range corrected GGA functional with RI-CC2 level as reference for molecules 35-46.

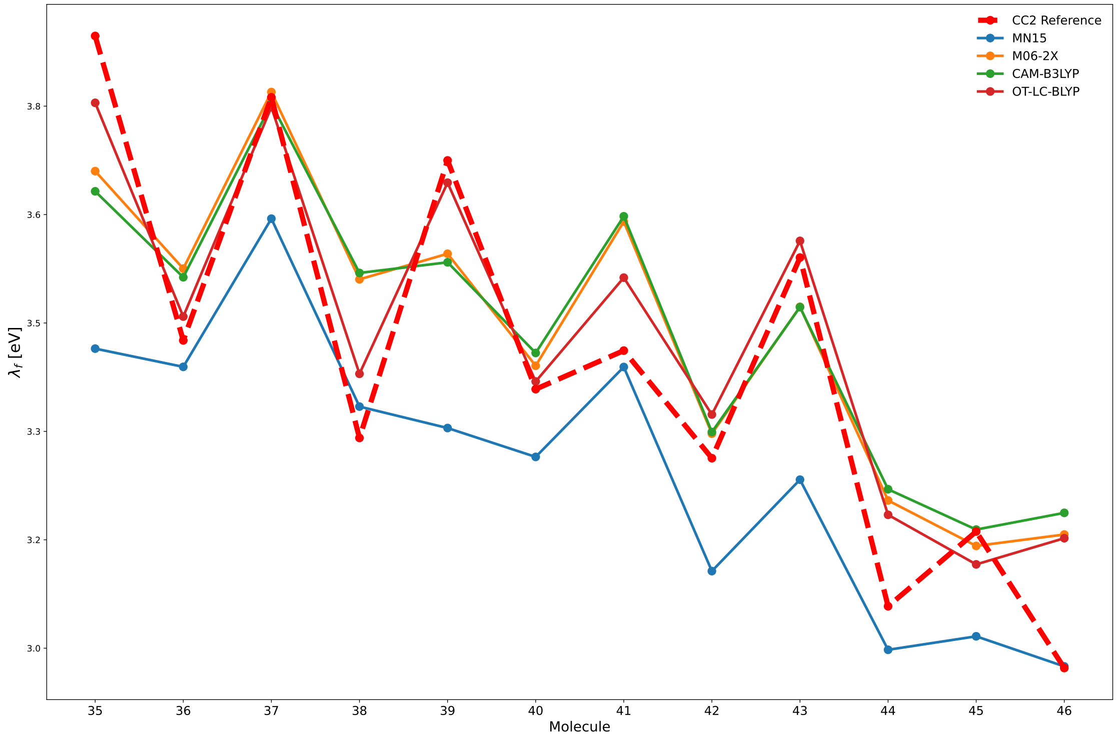

(b)

Figure S3: Excitation energy predictions for a selection of global hybid meta-GGA functionals and a long-range corrected GGA functional with RI-CC2 level as reference for molecules 35-46.

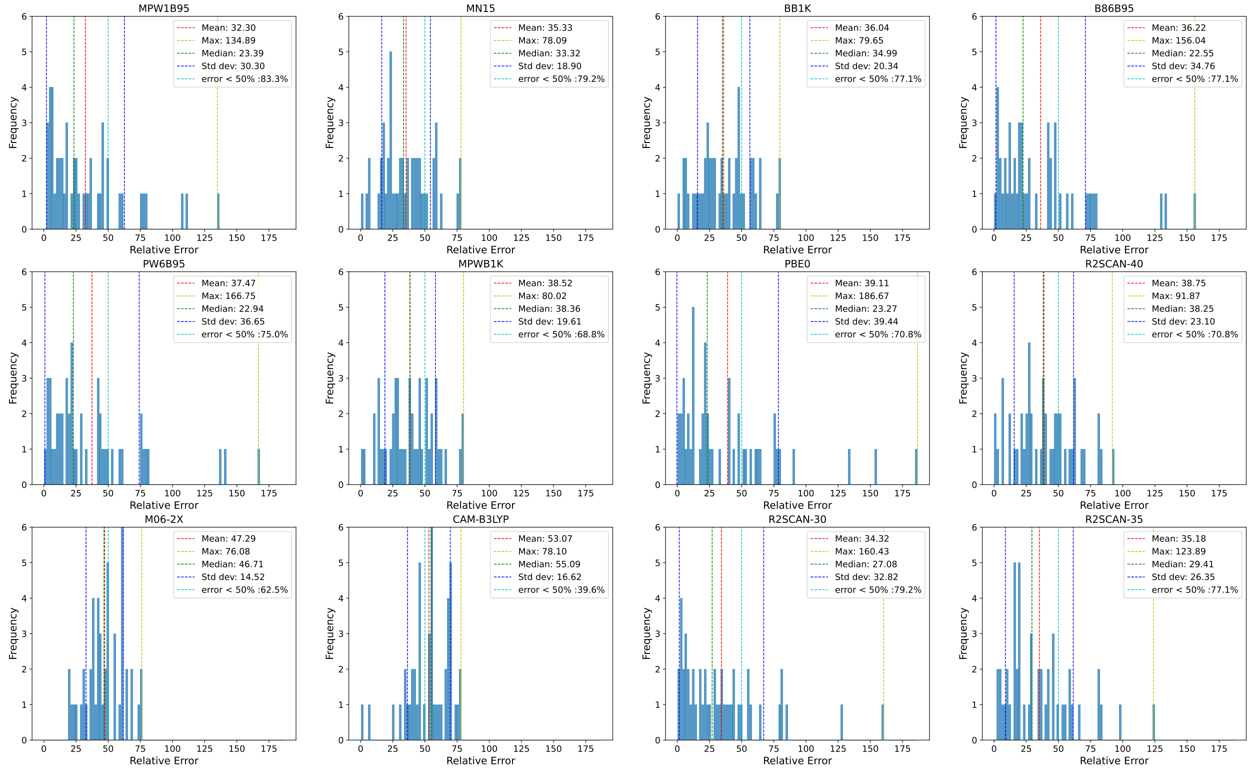

Figure S4: Distribution of relative errors for the calculation of 2PA strengths from the residue of quadratic response for molecules 1-48 with CC2 as reference.

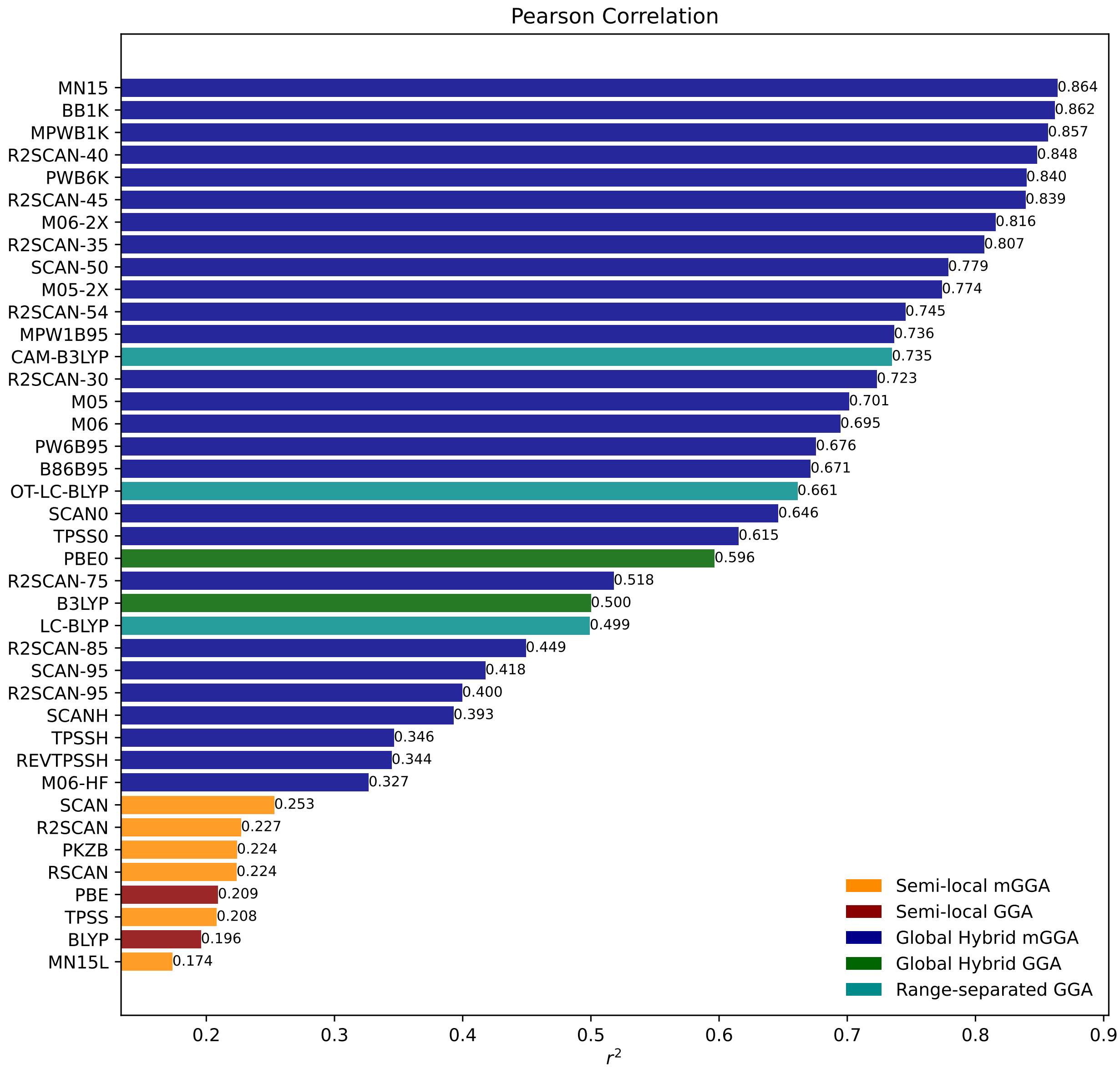

Figure S5: Pearson-Correlation (r^2) for the calculation of excited-state dipole moments for molecules 35-46 at the DFT level of theory with RI-CC2 as reference.

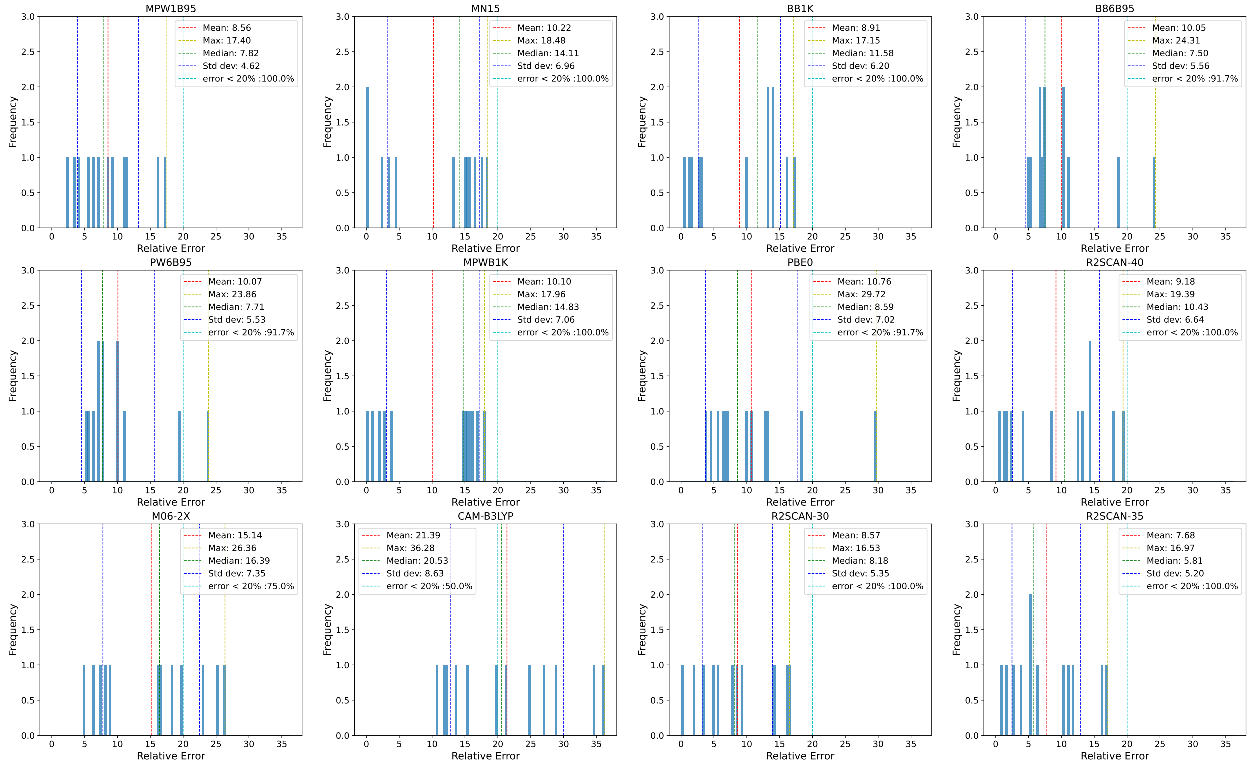

Figure S6: Distribution of relative errors for the calculation of excited-state dipole moments at the DFT and RI-CC2 level of theory for molecules 35-46.

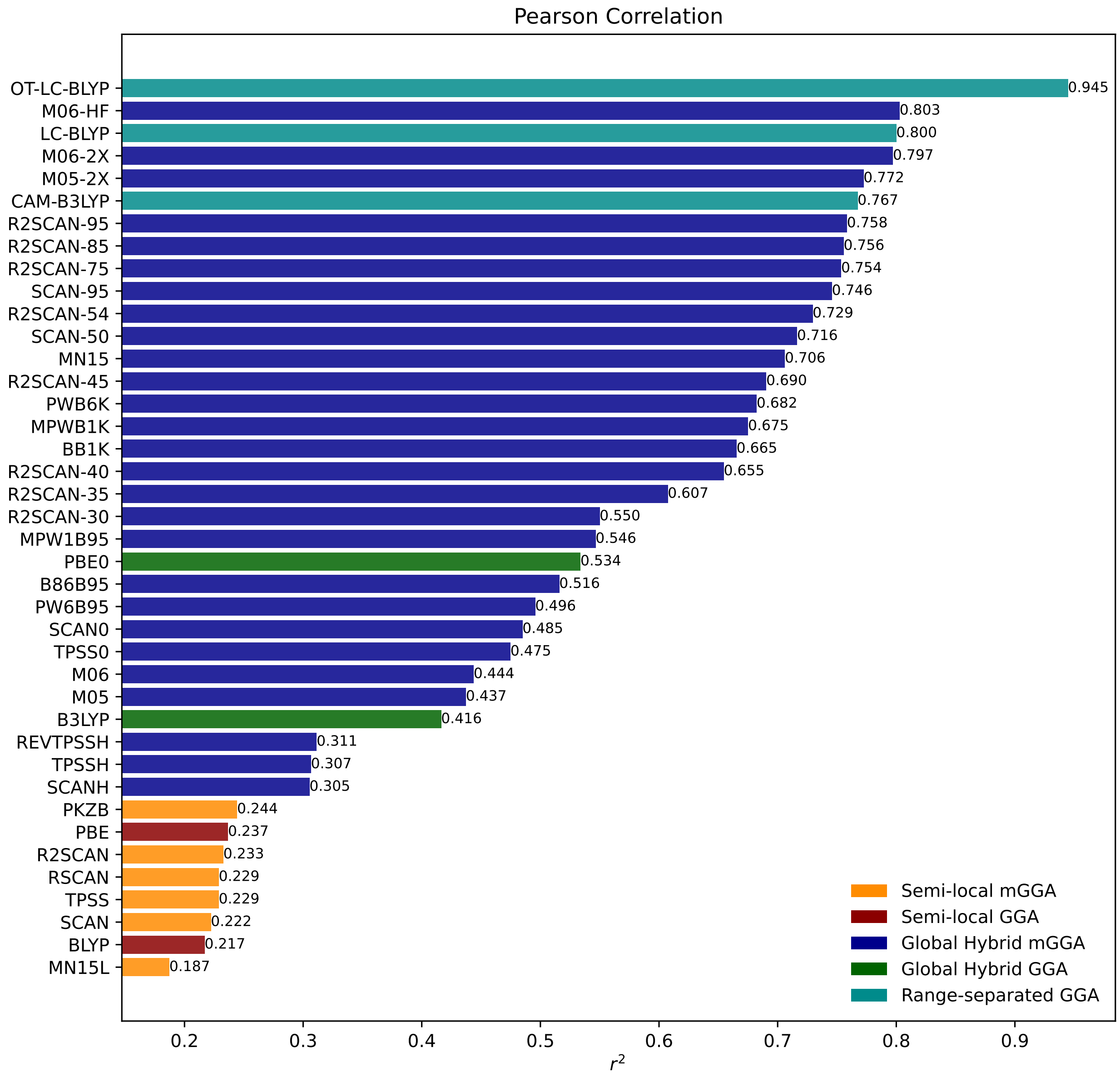

Figure S7: Pearson-Correlation (r^2) for the calculation of excitation energies for molecules 35-46 at the DFT level of theory with RI-CC2 as reference.

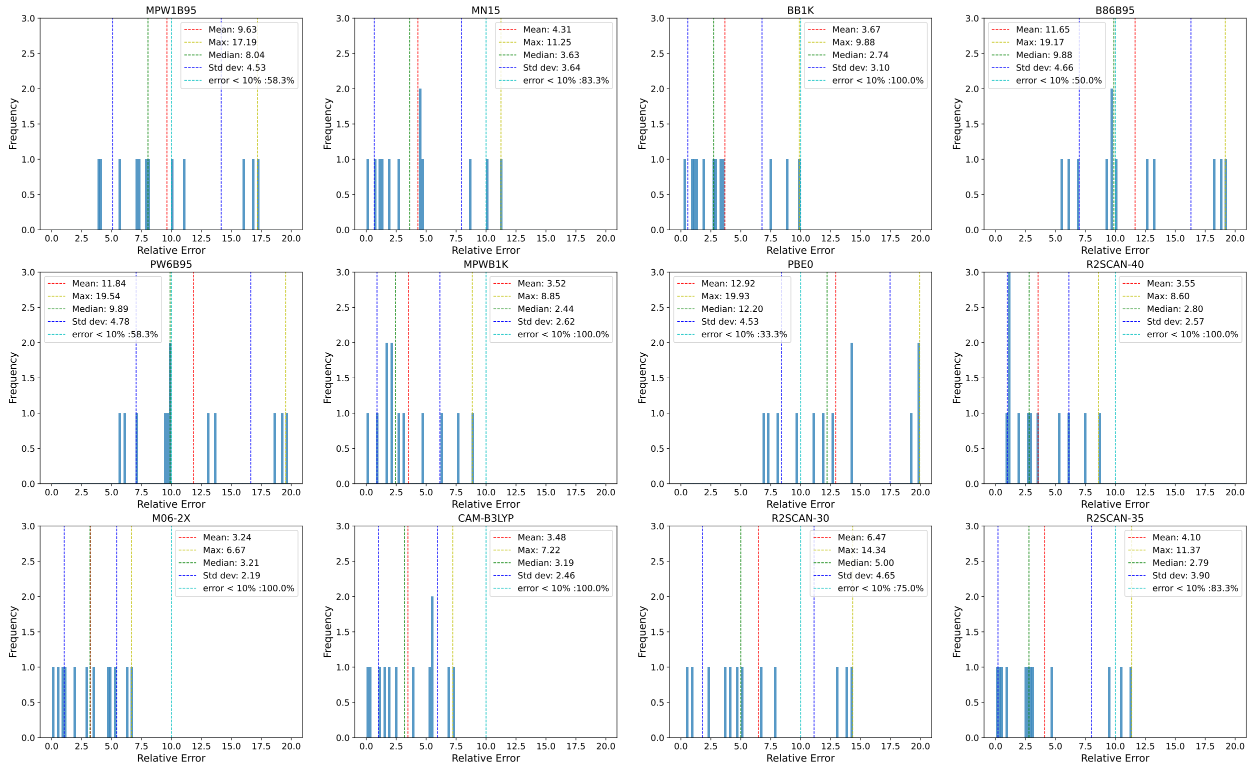

Figure S8: Distribution of relative errors for the calculation of excitation energies at the DFT and RI-CC2 level of theory for molecules 35-46.

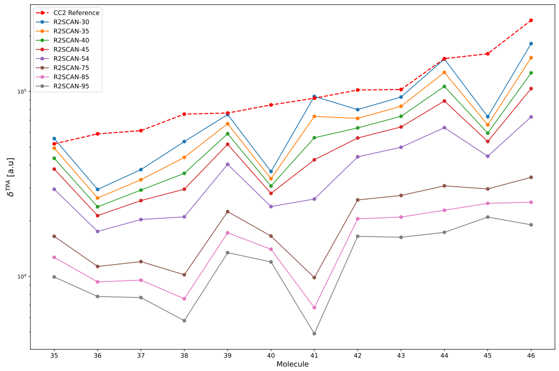

(a)

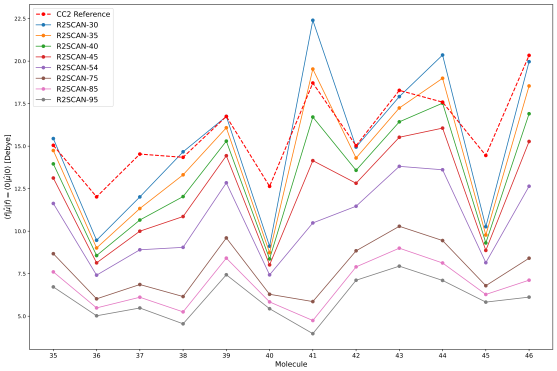

(b)

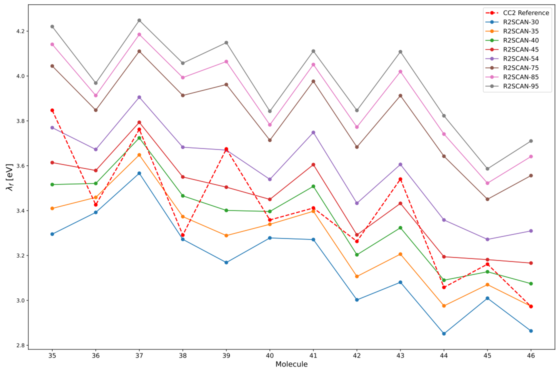

(c)

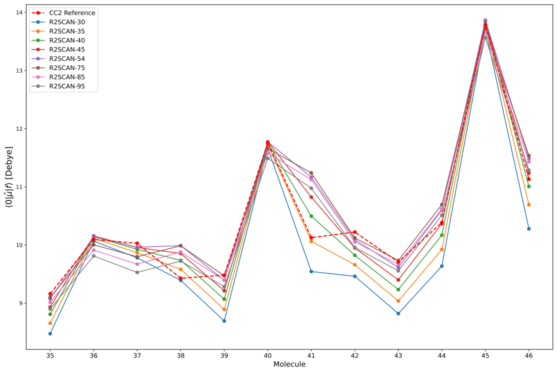

(d)

Figure S9: Illustration of the effect of the fraction of exact exchange on a) RSP 2PA strengths, b) difference between the excited- and ground-state dipole moments, c) excitation energies d), transition dipole moments.

Table S1: Mean relative error for the calculation of two-photon absorption strengths with the residue of the quadaratic response function. Reference: RI-CC2

Functional	Mean Relative Error	Mean Signed Relative Error	Min Relative Error	Max Relative Error
MPW1B95	32.30	-0.58	2.12 (9)	134.89 (1)
R2SCAN-30	34.32	-6.87	0.56 (44)	160.43 (1)
R2SCAN-35	35.18	-19.60	3.09 (15)	123.89 (1)
MN15	35.33	-32.48	0.64 (10)	78.09 (13)
BB1K	36.04	-30.06	1.86 (10)	79.65 (4)
B86B95	36.22	8.22	0.61 (31)	156.04 (1)
PW6B95	37.47	9.34	0.17 (31)	166.75 (1)
MPWB1K	38.52	-33.97	0.31 (32)	80.02 (4)
R2SCAN-40	38.75	-30.70	1.05 (10)	91.87 (1)
PBE0	39.11	12.03	0.28 (31)	186.67 (1)
SCAN0	39.14	0.14	0.22 (28)	208.47 (1)
M05	39.90	1.44	1.64 (43)	185.08 (1)
TPSS0	40.24	12.00	0.06 (31)	202.44 (1)
M06	41.53	4.79	0.80 (43)	206.14 (1)
PWB6K	41.66	-38.07	5.23 (2)	80.60 (4)
R2SCAN-45	44.88	-40.30	1.97 (2)	83.53 (4)
M06-2X	47.29	-47.29	19.44 (1)	76.08 (4)
M05-2X	48.81	-48.81	12.28 (1)	79.39 (4)
CAM-B3LYP	53.07	-53.05	0.45 (1)	78.10 (13)
SCAN-50	53.27	-50.36	23.41 (2)	84.73 (4)
R2SCAN-54	55.64	-54.15	11.05 (5)	84.46 (4)
B3LYP	56.25	32.97	1.56 (42)	262.93 (1)
OT-LC-BLYP	62.90	-62.90	41.13 (2)	76.75 (38)
LC-BLYP	71.51	-71.51	58.49 (15)	87.05 (41)
SCANH	73.12	49.22	0.48 (42)	352.72 (1)

Table S2: Mean relative error for the calculation of excited state-dipole moment from the double residue of the quadaratic response function. Reference: RI-CC2

Functional	Mean Relative Error	Mean Signed Relative Error	Min Relative Error	Max Relative Error
R2SCAN-35	7.68	-3.57	0.84 (43)	16.97 (45)
MPW1B95	8.56	2.19	2.22 (38)	17.40 (41)
R2SCAN-30	8.57	0.98	0.16 (46)	16.53 (41)
BB1K	8.91	-8.17	0.65 (39)	17.15 (45)
R2SCAN-40	9.18	-8.26	0.51 (39)	19.39 (45)
M06	9.40	0.92	0.23 (46)	18.09 (41)
M05	9.55	0.77	1.02 (38)	18.15 (44)
B86B95	10.05	4.43	5.01 (43)	24.31 (41)
PW6B95	10.07	4.52	5.18 (43)	23.86 (41)
MPWB1K	10.10	-9.95	0.01 (35)	17.96 (45)
SCAN0	10.18	1.75	1.86 (46)	22.53 (41)
MN15	10.22	-10.22	0.36 (35)	18.48 (45)
PBE0	10.76	4.74	3.65 (37)	29.72 (41)
TPSS0	10.93	5.61	4.54 (46)	28.86 (41)
PWB6K	12.03	-12.03	1.60 (35)	19.77 (41)
B3LYP	12.39	7.59	1.81 (37)	38.38 (41)
R2SCAN-45	12.84	-12.84	2.02 (44)	21.66 (45)
PBE	13.15	11.52	0.68 (43)	52.26 (41)
BLYP	13.24	11.63	0.62 (36)	52.89 (41)
TPSS	14.16	13.70	0.54 (45)	54.43 (41)
PKZB	14.18	12.95	0.96 (43)	53.61 (41)
SCANH	14.83	11.67	0.19 (37)	48.47 (41)
RSCAN	15.05	14.46	1.01 (45)	55.34 (41)
SCAN	15.09	13.99	0.19 (36)	54.53 (41)
M06-2X	15.14	-15.14	4.88 (35)	26.36 (41)
R2SCAN	15.18	14.59	0.99 (45)	55.42 (41)
TPSSH	15.21	13.93	0.33 (36)	51.20 (41)

Table S3: Mean relative error for the calculation of excitation energies. Reference: RI-CC2

Functional	Mean Relative Error	Mean Signed Relative Error	Min Relative Error	Max Relative Error
OT-LC-BLYP	2.05	1.21	0.32 (40)	6.04 (46)
M06-2X	3.24	1.42	0.19 (37)	6.67 (38)
CAM-B3LYP	3.48	1.54	0.09 (45)	7.22 (46)
M05-2X	3.52	1.81	0.29 (45)	7.22 (38)
MPWB1K	3.52	-1.29	0.12 (40)	8.85 (35)
PWB6K	3.54	-0.18	0.66 (40)	7.80 (35)
R2SCAN-40	3.55	-0.80	1.02 (37)	8.60 (35)
BB1K	3.67	-2.35	0.37 (44)	9.88 (35)
R2SCAN-45	3.99	1.69	0.64 (45)	7.88 (38)
R2SCAN-35	4.10	-3.52	0.04 (46)	11.37 (35)
MN15	4.31	-4.08	0.08 (46)	11.25 (35)
SCAN-50	4.82	3.66	0.48 (43)	9.81 (38)
R2SCAN-54	6.00	5.64	0.14 (39)	11.90 (38)
R2SCAN-30	6.47	-6.47	0.58 (38)	14.34 (35)
LC-BLYP	8.04	8.04	1.33 (35)	15.25 (46)
SCAN0	9.58	-9.58	3.29 (36)	17.65 (35)
MPW1B95	9.63	-9.63	3.96 (38)	17.19 (35)
TPSS0	11.06	-11.06	4.27 (36)	18.97 (35)
B86B95	11.65	-11.65	5.57 (36)	19.17 (35)
PW6B95	11.84	-11.84	5.62 (36)	19.54 (35)
M05	12.28	-12.28	5.97 (36)	20.62 (35)
R2SCAN-75	12.65	12.65	5.13 (35)	19.63 (46)
M06	12.81	-12.81	6.84 (36)	21.03 (35)
PBE0	12.92	-12.92	7.00 (36)	19.93 (35)
M06-HF	13.94	13.94	8.98 (35)	20.47 (46)
R2SCAN-85	15.15	15.15	7.64 (35)	22.49 (46)
B3LYP	17.14	-17.14	9.90 (36)	24.64 (39)

References

(S1) Rinkevicius, Z.; Li, X.; Vahtras, O.; Ahmadzadeh, K.; Brand, M.; Ringholm, M.; List, N. H.; Scheurer, M.; Scott, M.; Dreuw, A., et al. VeloxChem: A Python-driven density-functional theory program for spectroscopy simulations in high-performance computing environments. *WIREs* **2020**, *10*, e1457.
